# Spatial-Temporal Changes of Bacterioplankton Community along an Exhorheic River

**DOI:** 10.3389/fmicb.2016.00250

**Published:** 2016-03-03

**Authors:** Lili Ma, Guannan Mao, Jie Liu, Guanghai Gao, Changliang Zou, Mark G. Bartlam, Yingying Wang

**Affiliations:** ^1^Key Laboratory of Pollution Processes and Environmental Criteria (Ministry of Education), Tianjin Key Laboratory of Environmental Remediation and Pollution Control, College of Environmental Science and Engineering, Nankai UniversityTianjin, China; ^2^Department of Environmental Engineering and Safety Engineering, College of Chemistry and Chemical Engineering, Southwest Petroleum UniversityChengdu, China; ^3^LPMC and Institute of Statistics, Nankai UniversityTianjin, China; ^4^State Key Laboratory of Medicinal Chemical Biology, College of Life Sciences, Nankai UniversityTianjin, China

**Keywords:** bacterioplankton community, bacterial diversity, spatial pattern, seasonal pattern

## Abstract

To date, few aquatic microbial ecology studies have discussed the variability of the microbial community in exorheic river ecosystems on both the spatial and seasonal scales. In this study, we examined the spatio-temporal variation of bacterioplankton community composition in an anthropogenically influenced exorheic river, the Haihe River in Tianjin, China, using pyrosequencing analysis of 16S rRNA genes. It was verified by one-way ANOVA that the spatial variability of the bacterioplankton community composition over the whole river was stronger than the seasonal variation. Salinity was a major factor leading to spatial differentiation of the microbial community structure into riverine and estuarial parts. A high temperature influence on the seasonal bacterial community variation was only apparent within certain kinds of environments (e.g., the riverine part). Bacterial community richness and diversity both exhibited significant spatial changes, and their seasonal variations were completely different in the two environments studied here. Furthermore, riverine bacterial community assemblages were subdivided into urban and rural groups due to changes in the nutritional state of the river. In addition, the nutrient-loving group including *Limnohabitans, Hydrogenophaga*, and *Polynucleobacter* were abundant in the urbanized Haihe River, indicating the environmental factors in these anthropogenic waterbodies heavily influence the core freshwater community composition.

## Introduction

Microbial communities are key components in aquatic ecosystems and are among the most important players in biogeochemical cycling of fundamental elements (Azam and Worden, 2004). Learning about spatio-temporal changes in microbial populations associated with environmental parameters is an important means of understanding microbial ecology, as it provides insights into microbial distribution and their responses to environmental changes (Chapin et al., 2000; Fuhrman et al., 2006; Andersson et al., 2010). It is becoming increasingly clear that bacterioplankton communities can be highly dynamic across or within freshwater systems (Hiorns et al., 1997; Sekiguchi et al., 2002; Portillo et al., 2012; Liu et al., 2013). It has been suggested that bacterial community composition follows annually reoccurring patterns and exhibits predictable temporal patterns within a single environment (Yannarell et al., 2003; Portillo et al., 2012).

Spatial and temporal variations in a bacterioplankton community may often be attributed to a response to environmental changes. Factors such as water temperature (Crump and Hobbie, 2005; Kan et al., 2007), lake retention time (Lindström et al., 2005), salt concentration (Zhang et al., 2012), phytoplankton succession (Pinhassi et al., 2004; Niu et al., 2011), and the biomass of grazers on bacterioplankon (Lindström, 2000) have been suggested to be related to the bacterial community composition. Several studies have described shifts in bacterial community composition along aquatic salinity gradients and some have compared the composition of prokaryote communities in transition areas between freshwater systems and coastal marine environments (Crump et al., 1999; Cottrell and Kirchman, 2004; Henriques et al., 2006; Levipan et al., 2012). In addition to salinity, nutrients, and transparency have also been observed to influence the spatial variation of the aquatic microbial community composition (Lozupone and Knight, 2007; Fortunato et al., 2012; Liu et al., 2013). It was recently suggested that microbial community structure and function in stream sediments are controlled by land-use practices (Gibbons et al., 2014). Seasonality of bacterial community structure in an urban disturbed river was found to be mainly regulated by temperature (Zhang et al., 2012). Similarly, obvious temperature effects were also found in other aquatic ecosystems such as lakes (Shade et al., 2007; Lymer et al., 2008), estuarine, and coastal waters (Jing and Liu, 2012), which suggests a potential climate influence on the seasonal pattern of bacterial succession (Crump and Hobbie, 2005; Kent et al., 2007).

Despite recent advances in the characterization of aquatic microbial communities, few studies of aquatic bacterial communities have addressed the variability on both the spatial and seasonal scales (Fortunato et al., 2012). Fortunato and colleagues showed that spatial variability overwhelms seasonal patterns in bacterioplankton communities across environmental gradients from river to deep ocean (Fortunato et al., 2012). Jones and colleagues found the highest similarity of bacterioplankton communities was appeared from sample collected within the same lake, followed by samples collected through time within the same lake, and finally different lakes (Jones et al., 2012). As for river ecosystems, most studies to date have opted to focus on estuaries (Hewson and Fuhrman, 2004; Henriques et al., 2006; Jing and Liu, 2012; Levipan et al., 2012; Campbell and Kirchman, 2013), but have overlooked the whole river system which continuously changes with the surrounding environment, especially seasonal changes of bacterial communities, beta diversity, and the species driving those changes in the freshwater transects. Our knowledge of succession patterns in bacterial community structure from exorheic river systems on both the spatial and seasonal scales is still limited, especially for anthropogenically disturbed aquatic systems.

The objectives of this work were to characterize and compare the changes in bacterial communities in an anthropogenically disturbed exorheic river on both the spatial and seasonal scales, and to identify the diversity change along with the species driving those changes. In addition to denaturing gradient gel electrophoresis (DGGE), pyrosequencing was employed and the obtained data was further processed using the QIIME software package to provide taxonomic information on the community components over an annual sampling period along the Haihe River, China. The spatial and temporal variations of bacterial communities along the Haihe River were analyzed and compared to identify the predominant influencing factors. The mainstream of the Haihe River in Tianjin, China, is 72 km long and runs through the city center, followed by agricultural areas, and finally discharges into the Bohai Sea. In the last few decades, the rapid growth of the human population and economic development has inevitably resulted in a deterioration of water quality, and eutrophication has become the main problem affecting the Haihe River. These factors make the Haihe River an ideal river system in which to study the dynamic properties of the bacterial community under the influence of both natural and anthropogenic forces.

## Materials and methods

### Study sites and sample collection

**Figure 1:**
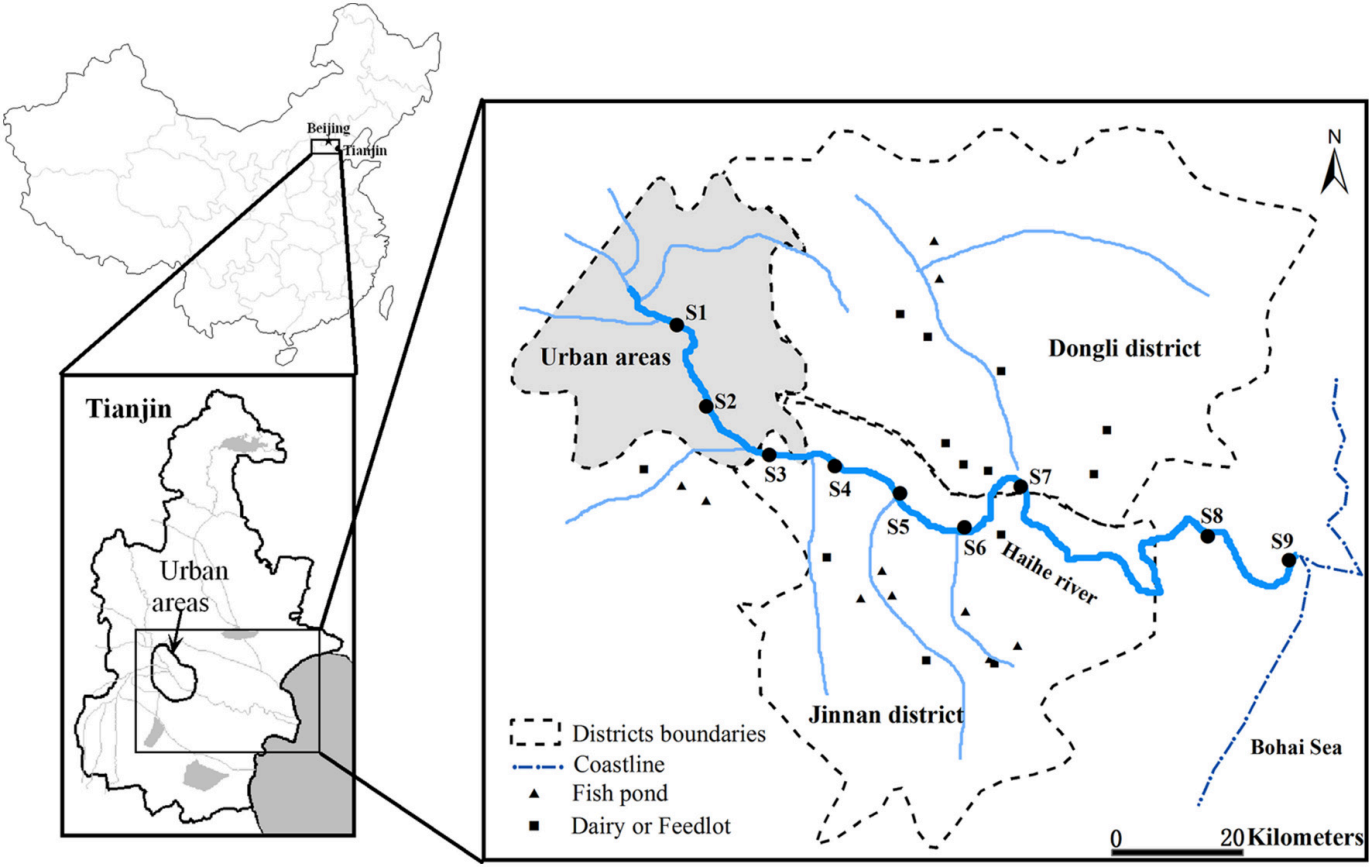
**Location of sampling sites along the Haihe River, China**. S1 and S2 were located in urban areas. S3–S7 were located in rural area adjacent to farmland, fish ponds, feedlots, and dairy farms. S8 and S9 were located at the river mouth of the Haihe River, at 8 and 1 km, respectively from the Bohai Sea.

Water samples were collected at three sections along the Haihe River (Figure 1) during the autumn (22th September) and winter (2nd December) of 2011, and spring (12th April) and summer (5th July) of 2012. For samples collected along the Haihe River, Sites 1 (S1) and 2 (S2) were located in urban areas with high population density. Sites 3 (S3)–7 (S7) were located in agriculturally influenced areas adjacent to farmland, fish ponds, feedlots and dairy farms. The remaining sample sites (S8 and S9) were located at the river mouth of the Haihe River, at 8 and 1 km respectively from the Bohai Sea. At each site, 2 L water samples were collected in clean, sterile bottles from a depth of about 0.5 m below the water surface. Water samples were kept cool during transport and stored at 4°C immediately upon arrival to the laboratory for 1–6 h until DNA extraction. Water samples for chemical analysis were collected at the same time in polyethylene terephthalate bottles which were rinsed three times with distilled water for laboratory analysis. With the exception of total suspended solids (TSS), total organic carbon (TOC), and chlorophyll a (chl a) content, which were analyzed as soon as possible after arrival to the laboratory, the samples for total nitrogen (TN), nitrate (NO_3_-N), total phosphorus (TP), total dissolved phosphorus (DTP) were acidified to pH < 2 using strong sulfuric acid. All samples were stored at 4°C before analysis and measured according to standard methods (Jin and Tu, 1990). Water temperature and salinity were measured on location by an YSI EC300 Water Quality Sonde.

### DNA extraction, PCR amplification and DGGE

Bacterial genomic DNA was extracted directly from the water sample using a Bacterial DNA kit (DNeasy, Qiagen, Germany) following the manufacturer's instructions. The protocols for PCR amplification and DGGE are based on those reported by Muyzer et al. (1993). A nested PCR technique was applied. The first PCR round employed the primers 63F and 1378R while the second round employed the primers 338F with a GC clamp at the 5′-end and 518R. During the first PCR round, 1 uL of DNA was added to 12.5 μL of PCR Master Mix (Promega, USA) with 0.5 μL of each primer, and then nuclease-free water was added into the mixture to make a final volume of 25 μL. In the second PCR round, the PCR reaction mixtures (50 μL) contained 1 μL of amplified product from the first round, 25 μL Colorless Master Mix (Promega, USA), 0.5 μL of each primer (10 μM), and 23 μL nuclear free water. Bacterial 16S rRNA genes for DGGE were amplified by a touch-down PCR program included a denaturing step at 94°C for 5 min, 10 touch-down cycles at 94°C for 60 s, 65–55°C for 60 s (−1°C per cycle), and 72°C for 60 s, 25 cycles at 94°C for 60 s, 55°C for 60 s, and 72°C for 60 s, and a final extension step of 72°C for 10 min. DGGE was performed using the Bio-Rad Decode universal mutation detection system (Bio-Rad, USA). Twenty microliter PCR products were loaded onto 8% (wt/*V*) polyacrylamide gels in 1 × TAE (40 mmol/L Tris, 20 mmol/L acetate, 1.0 mmol/L Na_2_-EDTA) with a denaturant-gradient of 30–60% (100% denaturant was 7 mol/L urea and 40% (wt/*V*) deionized formamide). The gels were electrophoresed at a constant voltage of 140 V and 60°C for 4 h. After electrophoresis, DGGE gels were stained with ethidium bromide (0.5 μg/mL) for 30 min in the dark. The stained gel was immediately photographed on a UV transillumination table with Image Quant 350 (GE Healthcare). The processing of the DGGE gels was performed with the software Quantity One (version 4.3, Bio-Rad, USA).

### PCR amplification and pyrosequencing

Eighteen samples were selected for high-throughput sequencing on the Roche 454 platform. Sampling dates were chosen to cover four seasons and sampling sites included urban (S2), rural (S5), and estuarial (S9) areas of the Haihe River. The other six samples were from the remaining sites (S1, S3, S4, S6, S7, and S8) in spring. Bacterial 16S rRNA genes were amplified by using the universal forward primer 338F and a cocktail containing four versions of the 1046R reverse primer (Huber et al., 2007). Primer 338F carried a 7 or 8 bp molecular barcode specific for each sample followed by a 454FLX adaptor A at the 5′ end. Primer 1046R carried a 454FLX adaptor B at the 5′end. Using this primer pair, the bacterial hypervariable regions V3 and V4 of the 16S rRNA gene were completely PCR amplified. For 454 sequencing, the entire V3 region can be covered and some reads even covered the entire V3–V5 or V6 region. The replicate PCR reactions were performed as described by Portillo and colleagues (Portillo et al., 2012), using the following cycling parameters: 25 cycles (94°C, 30 s; 55°C, 30 s; 72°C, 50 s) after an initial denaturation 94°C, 4 min. The replicate PCR reactions were pooled, purified using the QIAquick PCR Purification kit (Qiagen, Hilden, Germany) and quantified using an Agilent 2100 Bioanalyzer. Finally, equal amounts of PCR product for each sample were combined and sent for 454-sequencing at the Tianjin Key Laboratory of Microbial Functional Genomics (Tianjin, China). Pyrosequencing was performed on the 454 GS20 platform following the detailed protocol described in Sogin et al. (2006).

The raw sequences were first processed using the GL FLX software (Roche) for sorting and to trim low-quality sequences and primers. The obtained pyrosequencing data were processed using the QIIME software package (Caporaso et al., 2010). Sequences were quality controlled using the Split_Libraries.py script with settings as follows: exclude reads outside bounds of 200 and 1000; reads with low quality scores: below 25; maximum homopolymer exceeds length 6, and those missing the 16S primer or with uncorrectable barcodes, and primer sequences subsequently trimmed from both the beginning and the end of each “good” read. To account for pyronoise, the remaining sequences were denoised using the denoiser.py script. Chimeric sequences were identified using ChimeraSlayer and removed (Haas et al., 2011). Sequences that passed quality-filtering were then used to generate the dataset. The sequencing yielded a total of 194443 reads, and after the quality control, which left 165720 reads for further analysis. The sequences were then aligned and clustered into operational taxonomic units (OTU) using the Pyrosequencing Pipeline at the Ribosomal Database Project (RDP) website http://rdp.cme.msu.edu/ (Maidak et al., 2001) using complete linkage clustering and 3% distance threshold. 16S rRNA gene sequences longer than 1200 nucleotides and with good pintail score were downloaded from the RDP release and formatted into a local BLAST database. Each 454 sequence (one per group of identical sequences) was BLASTN searched against the RDP database with default parameters and inherited the taxonomic annotation (down to genus level) of the best scoring RDP hit, fulfilling the criteria of >90% identity over an alignment of length >180 bp. If no such hit was found, the sequence was classified as “no match.” Shannon diversity indices and Chao1 richness estimates were performed in QIIME with the pyrosequencing data sets all normalized to the same number of reads (Schloss et al., 2009). The 454 sequences read have been archived in the NCBI Short Read Archive under accession number SRP053333.

### Statistical analysis

Principal component analysis (PCA) was applied to experimental data standardized through z-scale transformation conducted by SPSS software, version 13.0 for Windows (Chicago, USA). Hierarchical cluster analysis was applied to the environmental parameters after log transformation using Average Linkage (between groups) were also conducted by SPSS software.

For analysis of DGGE data, a matrix of similarities for the densitometric curves of the band patterns was calculated based on the Dice coefficients and used to perform moving-window analysis (MWA; Wittebolle et al., 2005) by plotting the correlation between adjacent sampling sites. The Shannon-Wiener Index (*H*) was calculated to assess the structural diversity of the bacterial community:

$$\begin{array}{l} H=-\sum \left(n_{i}∕N\right)\log\left(n_{i}∕N\right) \end{array}$$

where *n*_*i*_ is the height of the peak and *N* the sum of all peak heights of the densitometric curve.

For statistical analysis of the pyrosequencing data, the pairwise similarities among samples were calculated by the unweighted UniFrac distance matrix (Lozupone and Knight, 2005). Plots of principal coordinate analysis (PCoA) and cluster analysis were used to visualize spatio-temporal dynamics in community structure by using the unweighted UniFrac distance matrix.

To statistically determine which is the major factor (spatial or seasonal) influencing the dynamics in bacterial community composition (based on pyrosequencing results), a one-way ANOVA model was established to verify whether there is a significant difference between spatial variation and seasonal variation, and to establish which one is stronger. Bacterial community data were spatially divided into riverine and estuarial groups according to sampling sites, and were seasonally divided into four groups according to seasons, which are defined as:

$$\begin{array}{l} X_{ij}\left(k\right),i=1,\ldots ,4,j=1,\ldots ,3,k=1,\ldots ,p; \end{array}$$

The one-way ANOVA model was:

$$\begin{array}{l} X_{ij}(k)=S_{i}(k)+\varepsilon_{ij}(k)\\ \text{ or}\\ X_{ij}(k)=L_{j}(k)+\varepsilon_{ij}(k) \end{array}$$

Where *S*_*i*_ and *L*_*j*_ represent seasonal effects and spatial effects, respectively.

If there is no significant difference between spatial variation and seasonal variation, the sum of the squared residuals based on the one-way ANOVA model will not be significantly different. This was computed as

(1) $$\begin{array}{l} SSE_{s}\left(k\right)=\overset{4}{\underset{i=1}{\sum }}\overset{3}{\underset{j=1}{\sum }}{\left\{X_{ij}\left(k\right)-{\bar{X}}_{i}\left(k\right)\right\}}^{2} \end{array}$$

(2) $$\begin{array}{l} SSE_{L}\left(k\right)=\overset{2}{\underset{j=1}{\sum }}\overset{4}{\underset{i=1}{\sum }}{\left\{X_{ij}\left(k\right)-{\bar{X}}_{\left(1\right)}\left(k\right)\right\}}^{2}+\overset{4}{\underset{i=1}{\sum }}{\left\{X_{i3}\left(k\right)-{\bar{X}}_{\left(2\right)}\left(k\right)\right\}}^{2}, \end{array}$$

where

$$\begin{array}{lll} {\bar{X}}_{i}\left(k\right) & = & \frac{1}{3}\sum_{j=1}^{3}X_{ij}\left(k\right),\\ {\bar{X}}_{\left(1\right)}\left(k\right) & = & \frac{1}{8}\left\{\sum_{i=1}^{4}X_{i1}\left(k\right)+\sum_{i=1}^{4}X_{i2}\left(k\right)\right\}\text{and}\\ {\bar{X}}_{\left(2\right)}\left(k\right) & = & \frac{1}{4}\sum_{i=1}^{4}X_{i3}\left(k\right) \end{array}$$

When *p* → ∞, we have

(3) $$\begin{array}{l} T=\frac{\sum_{k=1}^{p}\left\{SSE_{L}\left(k\right)-SSE_{S}\left(k\right)\right\}-2p\frac{n-1}{n-3}}{\sqrt{4p\frac{{\left(n-1\right)}^{3}}{{\left(n-3\right)}^{2}\left(n-5\right)}}}\overset{d}{\to }N\left(0,1\right), \end{array}$$

If the *T*-value is low and *p* < 0.05, it demonstrates that the spatial variation pattern of bacterial community significantly overwhelms the seasonal variation pattern.

Relationships between bacterial community similarity (pyrosequencing data) and environmental parameters were investigated using BIOENV analysis as implemented in the PRIMER V5 software package (Clarke and Warwick, 2001). Environmental data across the sites with seasons were used in BIOENV analysis. Environmental data were logarithmically transformed to satisfy the requirements of normality and variance homogeneity for BIOENV analysis. Analysis of Similarity Statistics (ANOSIM) was calculated to test the significance of differences among the sampling groups by using PRIMER V5. Shannon and Chao1 indices were calculated in QIIME. Spearman rank correlation analysis between environmental parameters and diversity indices (Shannon and Chao1) were conducted by SPSS software.

## Results

### Environmental parameters

The spatial variation of environmental parameters is summarized in Table 1 and Figure S1. PCA of the entire data set revealed three PCs with eigenvalues of >1 that explained about 79.0% of the total variance in the environmental data set. The first PC accounting for 40.8% of the total variance was correlated (loading > 0.70) with TN, TP, and DTP, which represents the trophic state of the river. The second PC accounting for 22.7% of total variance was correlated with salinity and chla. The third PC accounting for 15.5% of the total variance was correlated with TOC (Table S1). The cluster analysis of samples according to these parameters resulted in three clusters at Dlink/Dmax × 25 < 15 (Figure S2). The results indicated that Cluster I included samples from the urban areas (S1 and S2), which are associated with high TOC and NO_3_-N concentration (Table 1). Cluster II included samples far from the city center and near to the estuary of the Haihe River into the Bohai sea (S8 and S9), and were mainly characterized by high salinity and chl a levels (Table 1). The remainder of the samples collected from within rural areas were grouped together (S3, S4, S5, S6, and S7), and have relatively higher TSS along with greater nutrient concentrations (TN, TP, and DTP). Spatial clustering of all sites based on the water physical and chemical index reveals that water quality is related to the surrounding environment.

**Table 1:** **Environmental data (mean ± sd) recorded for the Haihe River from September 2011 to July 2012**.

	**S1**	**S2**	**S3**	**S4**	**S5**	**S6**	**S7**	**S8**	**S9**
Temp (°C)	20.15±7.34	20.68±7.89	20.10±7.83	20.18±8.11	19.48±8.38	19.43±8.18	19.40±8.38	19.53±8.25	19.40±7.64
Salinity(%0)	1.50±0.58	2.25±0.50	2.00±0.82	2.25±0.50	1.75±0.50	2.25±0.50	2.25±0.96	9.25±4.57	13.25±6.80
TN(mg L^−1^)	4.67±1.37	5.63±0.77	4.83±0.94	6.20±1.87	6.21±2.36	6.28±2.03	6.39±2.69	5.77±1.77	6.27±2.18
NO_3_-N(mg L^−1^)	2.27±0.48	2.27±0.57	1.70±0.83	1.95±0.69	2.22±0.19	2.21±0.23	2.12±0.68	1.94±0.76	1.76±0.61
TP(mg L^−1^)	0.37±0.20	0.45±0.23	0.50±0.20	0.68±0.42	0.74±0.29	1.72±2.07	2.59±3.67	1.12±0.78	0.74±0.30
DTP(mg L^−1^)	0.25±0.12	0.30±0.17	0.30±0.17	0.40±0.17	0.43±0.13	0.45±0.18	0.51±0.25	0.39±0.15	0.41±0.24
TOC(mg L^−1^)	32.23±15.65	22.91±12.04	18.84±13.35	17.70±6.22	16.01±7.35	33.54±25.38	14.09±14.30	23.62±12.55	24.78±10.95
Chl a(μg L^−1^)	53.52±36.71	54.74±32.66	58.68±49.71	93.86±117.44	68.69±66.17	58.97±51.49	47.20±72.76	117.12±96.10	72.44±74.48
TSS(mg L^−1^)	3.68±4.23	2.81±1.67	10.62±6.23	30.78±46.33	21.87±32.71	14.24±12.84	12.86±5.99	11.24±8.17	14.23±7.69

### Bacterial DGGE profiles

**Figure 2:**
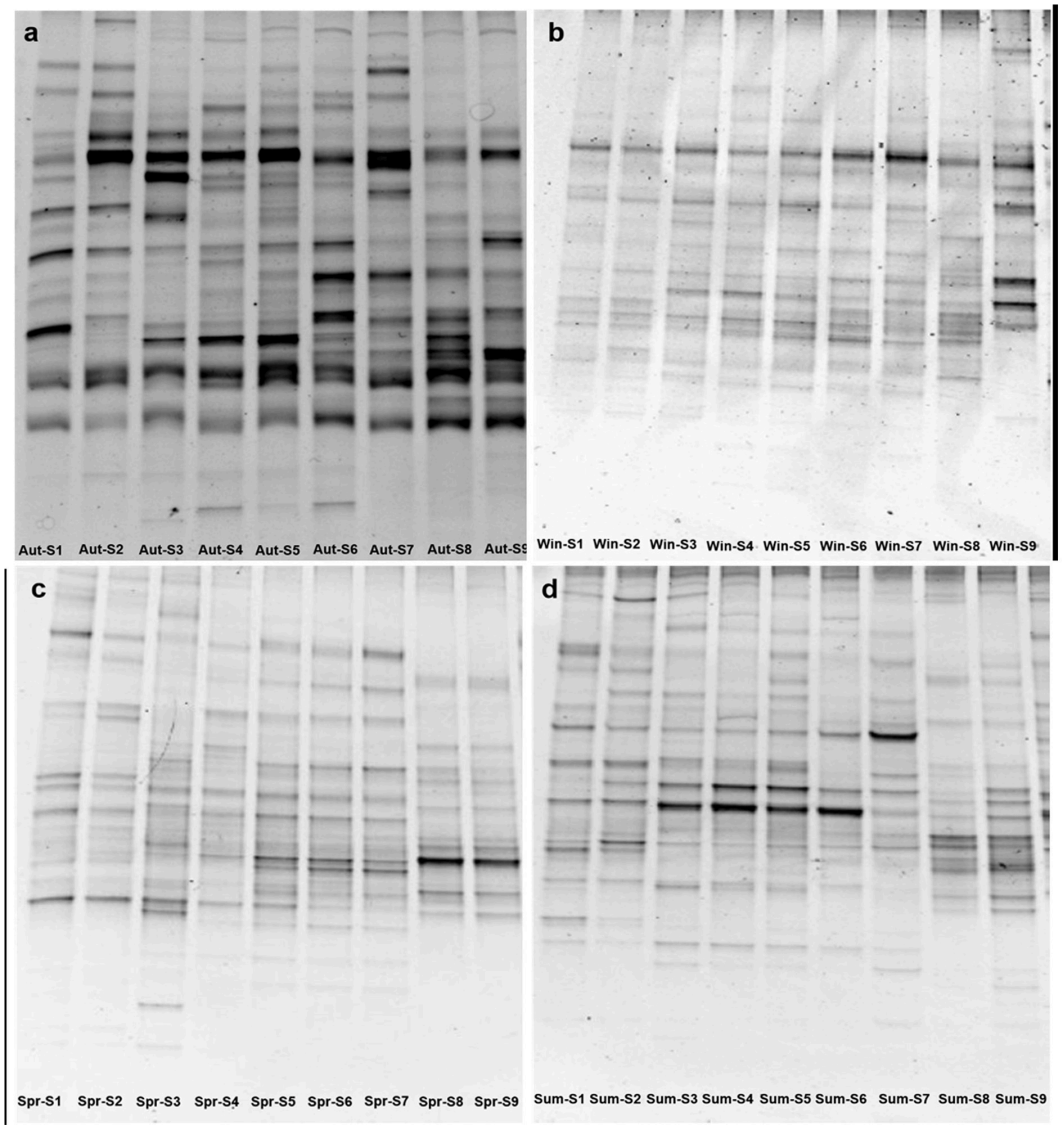
**Denaturing gradient gel electrophoresis fingerprints of 16S rRNA genes at nine sampling sites from September 2011 to July 2012, autumn (A), winter (B), spring (C), summer (D)**.

The DGGE patterns of bacterial communities for nine sampling sites along the river appeared to have obvious changes in each season (Figure 2). The number of DGGE bands detected per sample ranged from 12 to 28 in all samples investigated. Bacterial diversity, as determined by the Shannon index (*H*), fluctuated from 2.31 to 3.12, showing significant seasonal variation in the study (*P* < 0.05). As shown in Figure S3, the higher the changes between the DGGE profiles between adjacent sites, the lower the moving-window curve data point. In this study, two clear turning points were observed at S2–S3 and S7–S8 in the spring curve and autumn curve (Figure S3), suggesting spatial changes in bacterial communities between the urban and rural areas, and between the rural and estuarial areas along the Haihe River. The DGGE results indicated that there are seasonal and spatial (urban, rural, and estuarial areas) changes in bacterial communities along the Haihe River. In order to look more deeply into the factors driving the bacterial communities changes, 454 pyrosequencing was further employed.

### Spatio-temporal variation in bacterial community composition

**Figure 3:**
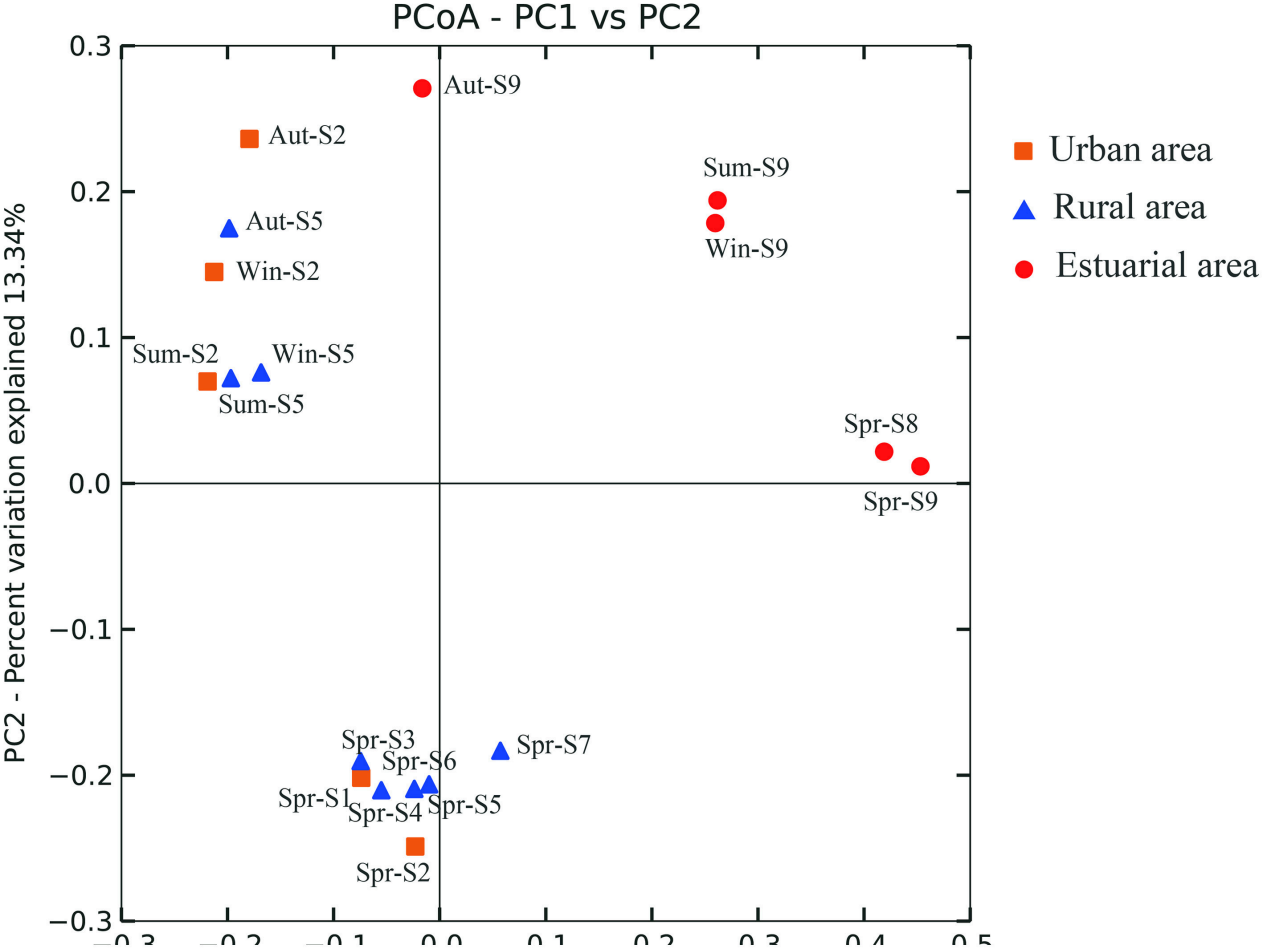
**Bacterial communities associated with different areas of the Haihe River by principal coordinate analysis (PCoA)**. Percentage of the diversity distribution explained by each axes is indicated on the figure. Winter samples (Win), autumn samples (Aut), summer samples (Sum) are shown for sites S2 (urban; orange), S4 (rural; blue), S8 (estuary; red). Spring samples (Spr) are shown for all sites.

**Figure 4:**
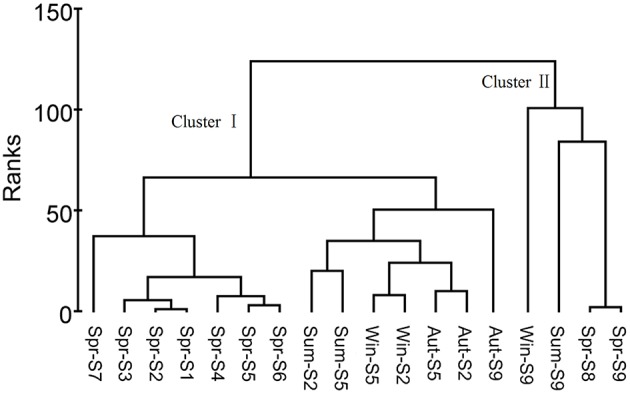
**Cluster analysis based on microbial community compositions in the Haihe River**. The cluster analysis of samples according to these parameters resulted in two clusters at ranks <150. Cluster I included samples from the urban and rural areas and Cluster II included samples from the estuary. Cluster I was subdivided into four groups according to seasons.

Based on the statistical analysis of pyrosequencing results, it was observed that estuarial bacterial communities formed clusters distinct from the riverine group (ANOSIM, *P* = 0.001; Figure 3). For the whole sampling area, seasonal variation appeared to be overwhelmed by the strong spatial variability. This hypothesis was further confirmed by the one-way ANOVA model. The *T*-value of -2.38 and the corresponding *P*-value of 0.0087 demonstrate that the spatial pattern of bacterial community composition is significantly stronger than the temporal changes. Seasonal variation became apparent in riverine sites (Figure 4). Riverine samples (sampled from sites 1 to 7) were grouped according to seasons, with samples from the same sampling season clustering together, independent of location (ANOSIM, *P* = 0.001; Figure 4). Furthermore, as shown in Figure 4, the spring samples along the Haihe River tend to form three clusters corresponding to the urban group, rural group, and estuarial group. This pattern was also observed from the community fingerprinting of 36 samples from April 2012 to July 2012 using DGGE (Figure S3). This suggests that the structure of bacterial communities exhibit differences between the three areas (urban area, rural area, and estuary), and the differentiation between urban and rural areas is less than between riverine and estuarial areas.

### Relationship between environmental variables and microbial community composition

To examine how environmental factors were related to the overall community composition, BIOENV analysis was performed based on the pyrosequencing results, to find the set of environmental parameters that, when combined, has the strongest correlation with the overall change in community composition (Clarke and Ainsworth, 1993; Table 2). The highest ranked correlation based on all sampling sites for all seasons was obtained with a combination of temperature and salinity (*r* = 0.759). When we focus only on riverine sites, temperature and TN were observed as factors most strongly correlated with bacterial community structures, with a correlation coefficient of 0.636. Moreover, the environmental influence on bacterial community variation was also detected within a single season. The BIOENV analysis based on spring data showed that the highest ranked correlation with community composition was obtained with the combination of TN, chl a, and salinity (*r* = 0.890).

**Table 2:** **Environmental influence on microbial community composition analyzed by BIOENV analysis**.

	**r**	**Environmental factor**
All sites, all seasons	0.759	TEMP, Salinity
	0.743	Salinity
	0.702	TEMP, chl a, Salinity
Riverine sites, all seasons	0.636	TEMP, TN
	0.601	TEMP, TSS, TN
	0.597	TEMP
Spring, all sites	0.890	TN, chl a, alinity
	0.875	DTP, Salinity
	0.869	TN, chl a, TP, Salinity

### Microbial biodiversity

**Figure 5:**
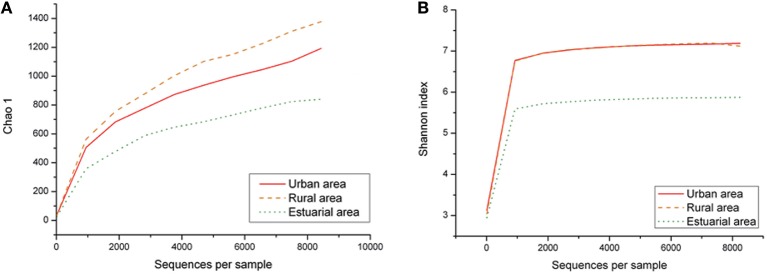
**Spatial variation of Chao 1 and Shannon index, Chao 1 (A), Shannon index (B)**.

Species richness according to the Chao1 index and the alpha diversity represented by the Shannon index origin from the pyrosequencing results, varied across the spatial groups, as shown in Figure 5. The richness of the entire bacterial community was significantly higher in the riverine group (rural and urban group, *p* < 0.05) than in the estuarial group. As with the shifts in bacterial community composition, we also observed different seasonal patterns in bacterioplankton richness and diversity levels between riverine and estuarial groups. The richness and diversity levels of the urban and rural groups were both lowest in autumn (*p* > 0.05). In contrast, the richness and diversity levels in the estuarial group were significantly different between autumn and winter (*p* < 0.05). It was showed the highest richness and diversity levels in autumn while the lowest occurred in winter. Moreover, we investigated the relationship between environmental factors and Chao1 index (out of 6415 sequences)/Shannon index (out of 6415 sequences). The correlation analysis revealed that species richness is significantly influenced by salinity over the entire study region (*r* = −0.61 *p* < 0.05). However, no significant correlation between bacterioplankton richness and other measured physicochemical factors was observed. Similar patterns were also observed for diversity values as measured by the Shannon index (*r* = −0.52 *p* < 0.05 for salinity).

### Composition of the river bacterial communities

**Figure 6:**
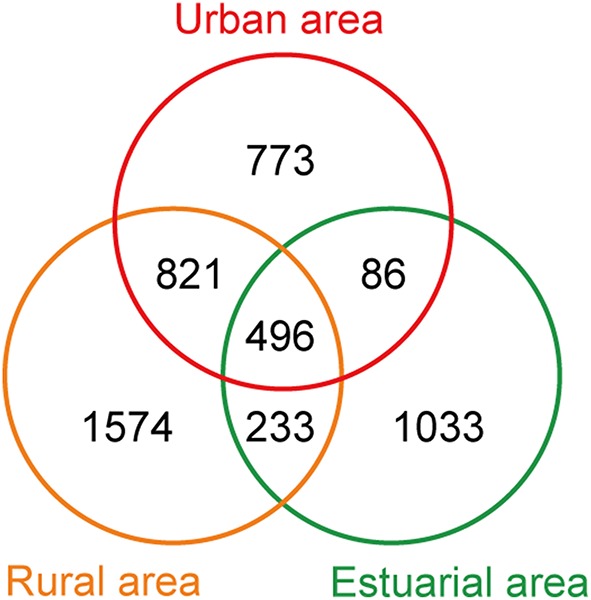
**Venn diagram of the number of OTUs from three different areas**.

**Figure 7:**
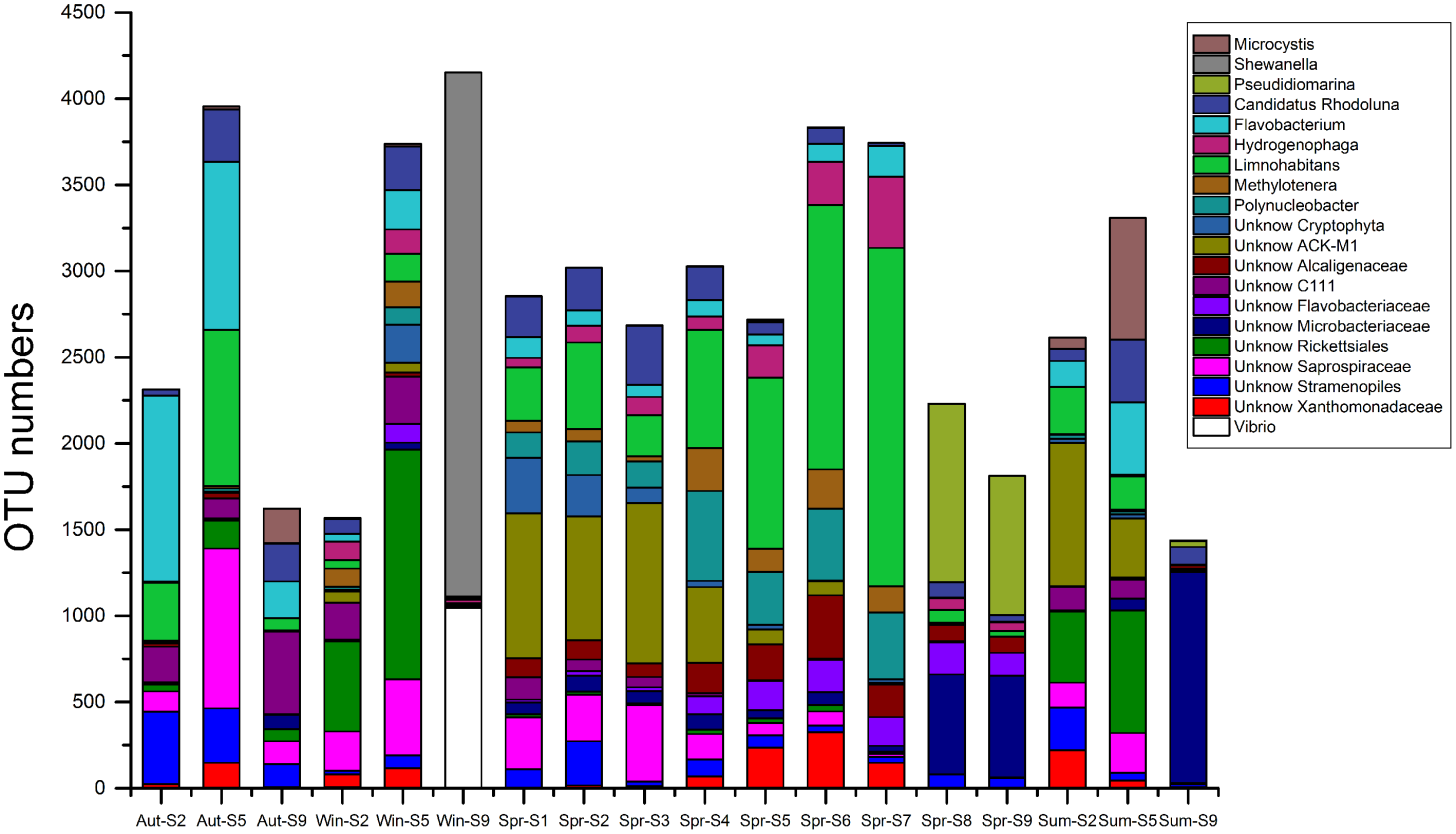
**The 20 most abundant genera for all samples in the Haihe River across four seasons**.

The 454-pyrosequencing data set consisted of 9206 ± 957 (average ± s.d.) sequences per sample, which were clustered into 5016 OTUs in total (97% cutoff). Of these sequences, more than 95% could be classified within Bacteria. In total, 496 OTUs were found in all three habitats, 1317 OTUs were shared by urban and rural communities, 3168 riverine OTUs were absent from estuary samples, and 1033 OTUs occurred exclusively in estuarial samples (Figure 6). The four most abundant phyla in the riverine group were Proteobacteria (43.5% including 17.4% Betaproteobacteria, 12.2% Alphaproteobacteria, 11.9% Gammaproteobacteria, and 2% Deltaproteobacteria), Bacteroidetes (25.7%), Actinobacteria (17.3%), and Cyanobacteria (9.3%). In the estuarial group, Proteobacteria (58.8% including 37.1% of Gammaproteobacteria, 14.8% of Alphaproteobacteria, 5.1% of Betaproteobacteria, and 1.6% of Deltaproteobacteria), Actinobacteria (16.8%), Bacteroidetes (10.7%), and Cyanobacteria (10.3%) were the four most abundant phyla. Spatio-temporal shifts in the microbial structure were analyzed at the genus level (Figure 7). An unclassified group at the genus level within *Stramenopiles* was evident in all sampling areas, but with higher abundance in riverine sites, especially in urban areas. The dominant genera in riverine sites were ACK-M1, *Limnohabitans*, unknown *Saprospiraceae, Hydrogenophaga, Flavobacterium*, and *Polynucleobacter*, which were less abundant in estuarial areas. The dominant genera in the estuarial area, unknown *Microbacteriaceae* and *Pseudidiomarina*, were minimally present in the riverine sites. In estuarial areas, the unknown *Microbacteriaceae* were abundant in spring and summer, but decreased in autumn. *Pseudidiomarina* was most abundant in spring. The genera *Vibrio* and *Shewanella* were only dominant in winter in the estuarial site. In riverine areas, *Microcystis* was most abundant in the summer samples. ACK-M1 was more abundant both in spring and summer, but less apparent in autumn and winter. *Flavobacterium* and unknown *Rickettsiales* were most abundant in autumn and winter, respectively.

## Discussion

It has been reported that the bacterial community in aquatic environments exhibited spatial patterns from rivers to the open sea (Troussellier et al., 2002; Hewson and Fuhrman, 2004) and short-term temporal shifts (e.g., annual cycle; Kent et al., 2004). However, most studies to date have focused mainly on spatial or seasonal scales, but rarely have they been assessed both spatially and temporally. In this study, we assessed bacterioplankton community composition of an anthropogenically disturbed river in both dimensions: spatially from river to estuary, and temporally in a 1-year time scale, from autumn to the following summer covering all four seasons.

Our data indicated that compared to seasonal changes, spatial variability in bacterioplankton communities was relatively stronger when the study area encompassed both riverine sites and estuarial sites, based on the relatively short time scale (1 year) study. This result was further verified by the ANOVA model that was first established here to directly validate which factor (seasonal or spatial) is stronger in regulating bacterioplankton community compositions. Seasonal bacterioplankton community changes overwhelmed by spatial changes were also evident in the marine ecosystem. Hatosy and colleagues reported that intra-seasonal temporal scales accounted for 73% of the community variation in marine bacteria (Hatosy et al., 2013). However, if we discard estuarial sites and only consider the riverine sites (Figure 4), a significant seasonal change was evident. These results are in line with a recent study in which bacterioplankton communities were separated into several distinct groups according to their habitats, and temporal variation was only detectable when each spatial group was analyzed separately (Fortunato et al., 2012). Differing spatio-temporal patterns of bacterioplankton community composition suggests that when the study area was restricted to a certain type of environment, temporal variability of bacterioplankton communities became obvious. Several studies of the bacterioplankton community composition from a specific habitat have shown repeatable seasonal changes, such as rivers (Tirodimos et al., 2010), estuaries (Fuhrman et al., 2006), coastal waters (Fuhrman et al., 2006), and the open ocean (Morris et al., 2005). In contrast, among different habitats, even among different parts of the same river, the spatial pattern of bacterioplankton communities may overwhelm the seasonal variation. Furthermore, as suggested by MWA (Figure S3) and clustering analysis (Figure 4), in addition to the estuarial group, bacterioplankton communities in the riverine sites are subdivided into an urban group and a rural group. This grouping pattern was also similar to the cluster pattern of environmental parameters. The reason may lie in the direct and indirect anthropogenic influence on bacterioplankton community compositions via the changing of river conditions caused by water pollution.

The spatio-temporal variability is often attributed to changes in key environmental factors that influence the rate of growth of individual populations. Salinity is known as a key contributor to microbial community structure and function (Lozupone and Knight, 2007; Nemergut et al., 2011). In estuarial and coastal environments, microbial communities commonly change in structure along salinity gradients (Campbell and Kirchman, 2013). In this study, salinity changed significantly between the river and estuarial areas (*p* < 0.05), and this factor appears to strongly influence the composition of bacterioplankton communities along the river to the estuary. When the data sets were analyzed based only on riverine sites, temperature, and TN rather than salinity were found to influence the community structure (Table 2). This suggests that salinity could be the primary environmental factor resulting in spatial differentiation in bacterioplankton communities from river to estuary along the Haihe River. These results are consistent with previous studies in which salinity were recognized as the most important environmental factors responsible for community composition (Fortunato et al., 2012). One plausible driver of seasonal change in bacterial community composition is temperature (Kan et al., 2007). Results from BIOENV analysis of riverine sites also suggest that temperature co-varies strongly with community composition (Table 2). Moreover, if we only extract and analyze the spring data, nutrients, chl a, and salinity were shown to influence the bacterioplankton community (Table 2). This observation was consistent with previous reports that nutrient concentrations can strongly affect bacterioplankton community composition, as the availability of phosphorus or nitrates may limit bacterioplankton growth in the aquatic environments (Cotner et al., 1997). Nutrients can also influence the phytoplankton and zooplankton compositions (Niu et al., 2011), and thus indirectly drive the changes of freshwater bacterioplankton community via food web dynamics involving both phyto- and zooplankton. In this study, the nutrient levels in the Haihe River usually increase when water flows through rural agricultural areas (Table 1). Agricultural phosphorus and nitrogen are considered to be important non-point sources of pollution of surface water in the Haihe River basin (Qiu et al., 2012; Sun et al., 2013). It has been reported inorganic N nutrients and certain other environmental factors caused by river discharges and associated contaminants would play important roles in the anammox process in the Bohai Sea (Dang et al., 2013). Therefore, land use influences (e.g., agricultural activities) on the microbial community structure via raising the essential nutrients for the growth of river microorganisms could not be ignored. Overall, the dynamics of bacterioplankton community composition in the Haihe River could possibly be influenced both by anthropogenic activities and natural drivers. The spatial pattern (along the river to the estuary) driven by salinity was stronger than seasonal variations (within a 1-year time scale) controlled by temperature, which is consistent with previous studies that show the major environmental determinant of microbial community composition is salinity rather than temperature, pH, or other physical and chemical factors (Lozupone and Knight, 2007).

Evenness, richness, phylogenetic diversity, and community structure were found to co-vary with one another in time (Shade et al., 2013). In the Haihe River, bacterial community richness and diversity were measured by the Chao1 and Shannon indices, respectively. Both the richness and Shannon index of the bacterial community exhibited the lowest values in the estuarial group, indicating that when water mixes from freshwater to saltwater, the number of taxa at the genera level is reduced (Figure 7). The loss of bacterial community along the salinity gradient could result from cellular deactivation and death of a proportion of freshwater bacteria that did not adapt to the increased salinity (Bouvier and del Giorgio, 2002; del Giorgio and Bouvier, 2002; Cottrell and Kirchman, 2004). Additionally, the bacterial diversity level in riverine sites was lowest in autumn, while in estuary sites it was highest in autumn. The seasonal variations of bacterioplankton diversity levels were completely different in these two studied environments. Meanwhile, in light of possible differences within each genera (Newton and McLellan, 2015), further studies will be focused on the more detailed changes at the species level.

The number of OTUs shared by urban and rural sites was greater than the number of OTUs shared by riverine sites and estuarial sites. The composition of bacterial communities showed a slight overlap among all three areas, and predominant groups are apparently different between riverine and estuarial environments. The two most abundant groups (Bacteroidetes and Betaproteobacteria) in studied riverine sites are typical for freshwater ecosystems (Zwart et al., 2002). Bacteroidetes were observed to correlate with high nutrient levels (Brümmer et al., 2000; de Figueiredo et al., 2007) and were usually abundant in mesotrophic and eutrophic water bodies (Riemann and Winding, 2001; Van der Gucht et al., 2005; de Figueiredo et al., 2007; Niu et al., 2011). Concordantly, Bacteroidetes were widely distributed in the Haihe River perhaps due to its eutrophic nature. The wide distribution of Betaproteobacteria in freshwater habitats may be due to their rapid response to addition of nutrient addition. The most abundant taxa in the riverine sites were belong to Comamonadaceae, containing the representatives of betI, which is a fast-growing and nutrient-loving group (Glöckner et al., 2000; Zwart et al., 2002). The existence of these nutrient-loving groups in riverine areas indicates that high nutrient concentration is one likely driver of changes in the bacterial assemblage in these urbanized water systems. Consistent with previous studies, many typical freshwater bacteria such as Actinobacteria ACK-M1, *Limnohabitans, Hydrogenophaga, and Polynucleobacter* (Zwart et al., 2002; Hahn, 2006; Wang et al., 2009) have also been observed in high abundance in both urban and rural areas. *Microcystis*, often regarded as the major contributor of cyanobacteria blooms (Chen et al., 2003), were only abundant in rural sampling sites. This may be due to the high level of phosphorus in rural agricultural areas, because *Microcystis* blooms are associated with high phosphorus concentrations (Harke and Gobler, 2013), and the growth rates of toxic populations of *Microcystis* was found to be significantly facilitated by phosphorus loading (Davis et al., 2009). These results were in accordance with a recent study which reported *Limnohabitans, Polynucleobacter*, and *Rhodobacter* had increased representation in urban-impacted waterbodies (Newton and McLellan, 2015). Gammaproteobacteria were most abundant in marine-freshwater transition areas in this study, which is consistent with previous studies on brackish waters, estuarine, and coastal waters (Bouvier and del Giorgio, 2002; Henriques et al., 2006; Jing and Liu, 2012). Most OTUs in riverine communities faded out in estuarial areas, as some dominant riverine bacteria are poor at surviving in seawater (Crump et al., 1999). A study of bacterial composition in Baltic Sea demonstrated that there are various combination of freshwater and marine clades in brackish waters, that appears to have adapted to the brackish conditions (Herlemann et al., 2011) This mixing of bacterial communities may also occur in the Haihe River estuary, where both typical freshwater taxa (like members of Betaproteobacteria) and marine water taxa (like *Pseudoalteromonas, Pseudidiomarina*, etc.) were present.

## Conclusions

In conclusion, this work represents one of the few detailed studies to date that characterize the changes in bacterial communities in an anthropogenically disturbed river. In this study, it was observed that (1) In the 1 year time scale, the spatial variability pattern in bacterioplankton community composition was stronger than the seasonal variation across the river to the estuary; (2) Both the richness and Shannon index of the bacterial community exhibited the lowest values in the estuarial group, and the seasonal pattern of beta-diversity was different in the river from urban areas to the estuary; (3) The nutrient-loving groups including *Limnohabitans, Hydrogenophaga*, and *Polynucleobacter* were abundant in the urbanized Haihe River, indicating the environmental factors in these anthropogenic waterbodies heavily influence the core freshwater community composition.

## Author contributions

MB and YW initiated and designed the research; LM, GM, and GG performed the research and collected samples; LM, LJ, and YW analyzed microbial diversity; LM, GM, GG, and CZ performed the statistical analysis; LM, GM, CZ, MB, and YW analyzed data; LM, GM, MB, and YW wrote the manuscript. All authors reviewed the manuscript.

### Conflict of interest statement

The authors declare that the research was conducted in the absence of any commercial or financial relationships that could be construed as a potential conflict of interest.
