# Supplementary material for: Spatial-Temporal Changes of Bacterioplankton Community along an Exhorheic River
Source: Front Microbiol. 2016 Mar 3;7:250. doi: 10.3389/fmicb.2016.00250 (PMC4776164; doi:10.3389/fmicb.2016.00250)
Supplement: Supplementary file 1 [file Table1.DOCX]

Supplementary Material

Spatial-temporal changes of bacterioplankton community along an exhorheic river

**Lili Ma, Guannan Mao, Liu Jie, Guanghai Gao, Changliang Zou, Mark Bartlam*, Yingying Wang**

*** Correspondence:** Yingying Wang Email: [wangyy@nankai.edu.cn](mailto:wangyy@nankai.edu.cn);

Mark Bartlam Email: [bartlam@nankai.edu.cn](mailto:bartlam@nankai.edu.cn);

# Supplementary Figures and Tables

## Supplementary Figures


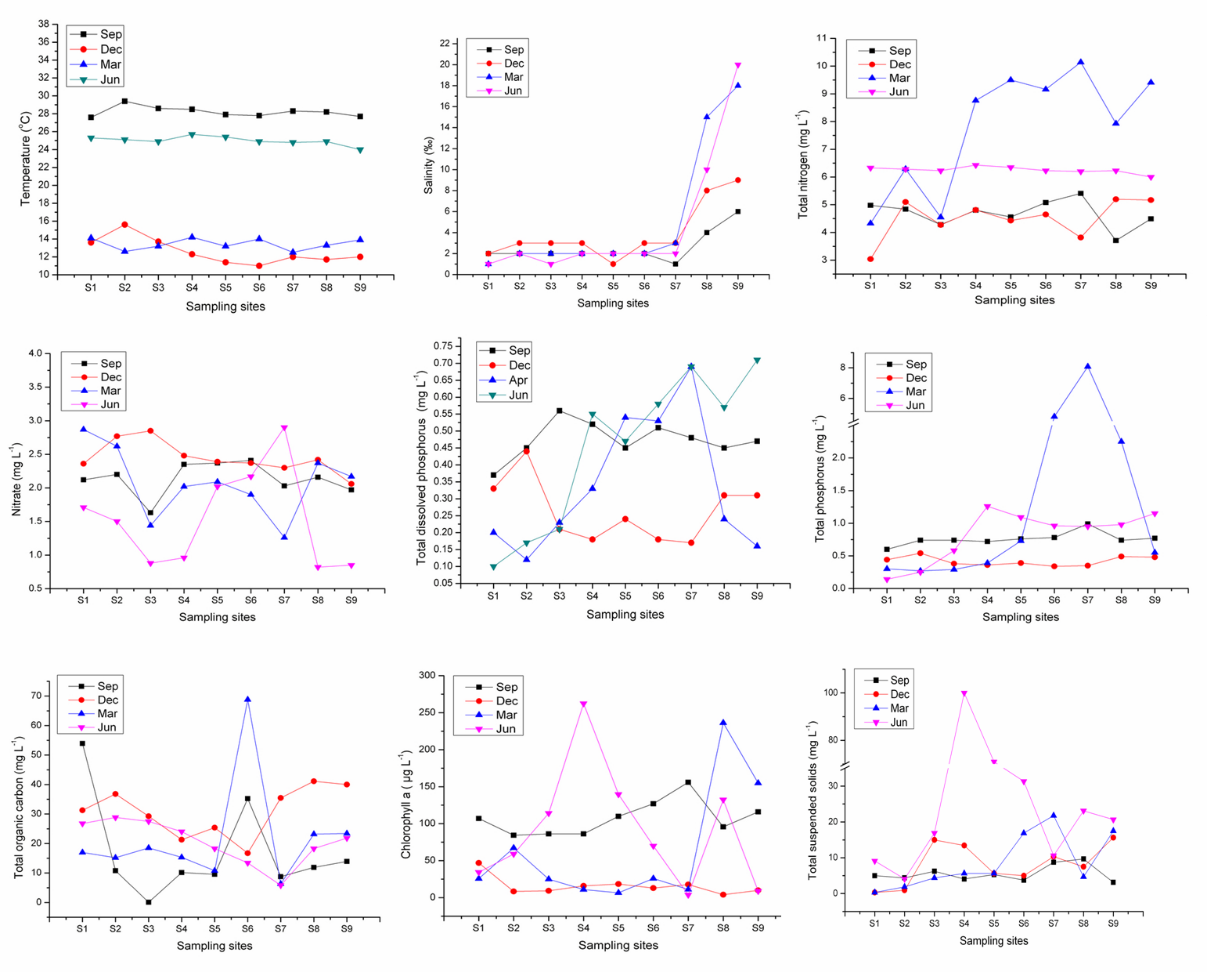


**Supplementary Figure S1.** Environmental data recorded for the Haihe River.

**
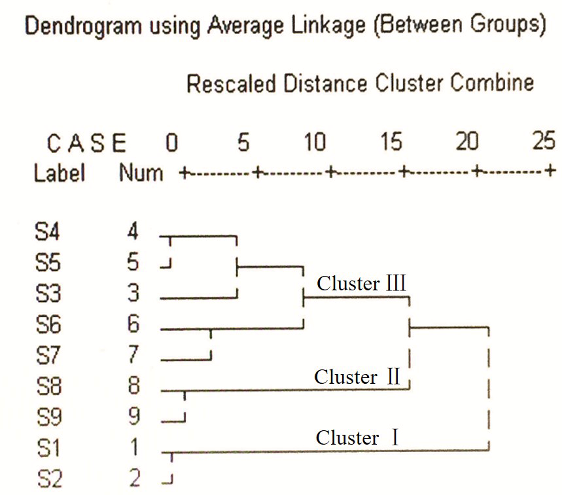
**

**Supplementary Figure S2.** Cluster analyses of environmental parameters in water samples of the Haihe River from September 2011 to July 2012. The cluster analysis of samples according to these parameters resulted in three clusters at Dlink/Dmax ×25 < 15. Cluster Ⅰ included samples from the urban areas (S1 and S2), Cluster Ⅱ included samples far from the city center and near to the estuary of the Haihe River into the Bohai sea (S8 and S9), and Cluster Ⅲ included samples from within rural areas (S4 and S7).


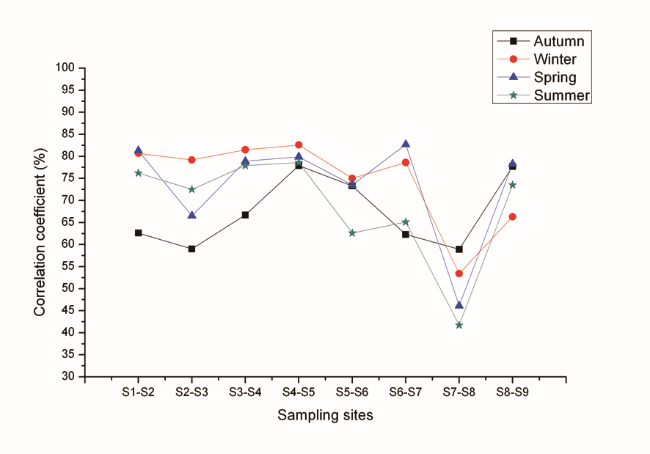


**Supplementary Figure S3.** Moving-window analysis based on DGGE profiles for adjacent sampling sites. The variability between two consecutive sites was calculated based on the denaturing gradient gel electrophoresis patterns as represented in Fig. 2 The higher the changes between the DGGE profiles of two consecutive sampling points, the lower the corresponding moving window curve data point will be.

## Supplementary Tables

**Supplementary Table S1. Loadings of experimental variables on significant principal components (with Varimax rotation) for the data set.**

| Variables | Component | | |
| --- | --- | --- | --- |
|  | 1 | 2 | 3 |
| Temp | -0.858 | -0.289 | 0.073 |
| Salinity | 0.256 | **0.860** | -0.292 |
| TN | **0.816** | 0.102 | 0.371 |
| NO_3_-N | 0.101 | -0.771 | -0.202 |
| TP | **0.867** | -0.263 | 0.026 |
| DTP | **0.937** | 0.026 | 0.344 |
| TOC | -0.088 | -0.001 | -0.831 |
| Chla | 0.003 | **0.760** | 0.243 |
| TSS | 0.282 | 0.251 | **0.800** |
| Eigenvalue | 3.672 | 2.044 | 1.393 |
| % Total variance | 40.8 | 22.7 | 15.5 |
| Cumulative % variance | 40.8 | 63.5 | 79.0 |
